# Hepatitis C Treatment Initiation Among US Medicaid Enrollees

**DOI:** 10.1001/jamanetworkopen.2023.27326

**Published:** 2023-08-04

**Authors:** Shashi N. Kapadia, Hao Zhang, Christopher J. Gonzalez, Bisakha Sen, Ricardo Franco, Kayla Hutchings, Elaine Wethington, Andrew Talal, Audrey Lloyd, Arpan Dharia, Martin Wells, Yuhua Bao, Martin F Shapiro

**Affiliations:** 1 Division of Infectious Diseases, Weill Cornell Medicine, New York, New York; 2 Department of Population Health Sciences, Weill Cornell Medicine, New York, New York; 3 Division of General Internal Medicine, Weill Cornell Medicine, New York, New York; 4 Department of Health Policy and Organization, University of Alabama at Birmingham, Birmingham; 5 Division of Infectious Diseases, University of Alabama at Birmingham; 6 Department of Sociology and Department of Psychology, Cornell University, Ithaca, New York; 7 Division of Gastroenterology, Hepatology, and Nutrition, Jacobs School of Medicine and Biomedical Sciences, University at Buffalo, Buffalo, New York; 8 Department of Statistics and Data Science, Cornell University, Ithaca, New York

## Abstract

**Importance:**

Direct-acting antiviral (DAA) treatment for hepatitis C virus (HCV) infection is highly effective but remains underused. Understanding disparities in the delivery of DAAs is important for HCV elimination planning and designing interventions to promote equitable treatment.

**Objective:**

To examine variations in the receipt of DAA in the 6 months following a new HCV diagnosis.

**Design, Setting, and Participants:**

This retrospective cohort study used national Medicaid claims from 2017 to 2019 from 50 states, Washington DC, and Puerto Rico. Individuals aged 18 to 64 years with a new diagnosis of HCV in 2018 were included. A new diagnosis was defined as a claim for an HCV RNA test followed by an *International Statistical Classification of Diseases and Related Health Problems, Tenth Revision* (*ICD-10*) diagnosis code, after a 1-year lookback period.

**Main Outcomes and Measures:**

Outcome was receipt of a DAA prescription within 6 months of diagnosis. Logistic regression was used to examine demographic factors and *ICD-10*–identified comorbidities associated with treatment initiation.

**Results:**

Among 87 652 individuals, 43 078 (49%) were females, 12 355 (14%) were age 18 to 29 years, 35 181 (40%) age 30 to 49, 51 282 (46%) were non-Hispanic White, and 48 840 (49%) had an injection drug use diagnosis. Of these individuals, 17 927 (20%) received DAAs within 6 months of their first HCV diagnosis. In the regression analyses, male sex was associated with increased treatment initiation (OR, 1.24; 95% CI, 1.16-1.33). Being age 18 to 29 years (OR, 0.65; 95% CI, 0.50-0.85) and injection drug use (OR, 0.84; 95% CI, 0.75-0.94) were associated with decreased treatment initiation. After adjustment for state fixed effects, Asian race (OR, 0.50; 95% CI, 0.40-0.64), American Indian or Alaska Native race (OR, 0.68; 95% CI, 0.55-0.84), and Hispanic ethnicity (OR, 0.81; 95% CI, 0.71-0.93) were associated with decreased treatment initiation. Adjustment for state Medicaid policy did not attenuate the racial or ethnic disparities.

**Conclusions:**

In this retrospective cohort study, HCV treatment initiation was low among Medicaid beneficiaries and varied by demographic characteristics and comorbidities. Interventions are needed to increase HCV treatment uptake among Medicaid beneficiaries and to address disparities in treatment among key populations, including younger individuals, females, individuals from minoritized racial and ethnic groups, and people who inject drugs.

## Key Points

**Question:**

Are there disparities in the initiation of hepatitis C treatment among Medicaid enrollees?

**Findings:**

In this retrospective cohort study of 87 652 US Medicaid enrollees, there was low treatment uptake for hepatitis C overall and significantly lower treatment initiation among people younger than 30 years, females, Hispanic and Asian individuals, and people with injection drug use.

**Meaning:**

These findings suggest that interventions are needed to increase treatment rates for hepatitis C overall and among key populations and ensure equity in treatment within the Medicaid program.

## Teaser

This cohort study examines variations in the receipt of direct-acting antiviral treatment in the 6 months following a new diagnosis for hepatitis C virus.

## Introduction

Hepatitis C virus (HCV) infection continues to be a major public health problem. From 2013 to 2020, the incidence of acute HCV doubled; 60% of cases are among persons aged 20 to 39 years, and 66% among people who inject drugs (PWID).^1^ Direct-acting antiviral (DAA) medications, available since 2014, represent a major therapeutic advancement. Their use has been shown in Medicare and Veterans’ Administration data to reduce racial and ethnic disparities in HCV treatment.^2,3^ Unfortunately, treatment uptake in the US has been insufficient to achieve HCV elimination by 2030.^4^

The slow treatment uptake, combined with changing HCV epidemiology, may exacerbate disparities. Whereas HCV was once concentrated in the baby boomer generation (ie, people born between 1945-1965), recent years have seen increasing infections in people younger than 40 years, driven largely by injection drug use (IDU).^1^ Treatment rates for young PWID have been low in smaller-scale studies.^5,6^ Although initially concentrated in nonurban and White populations, reported new HCV cases among Black populations have increased almost 4-fold between 2016 and 2020, the fastest rate of increase among individuals from minoritized racial and ethnic groups.^1^ Younger individuals with newly diagnosed HCV may have different patterns of health care access and priorities than older cohorts; thus, previous findings suggesting that disparities in treatment were ameliorated by DAAs may no longer apply.

Understanding treatment-related disparities in Medicaid is critical to elimination planning, since 55% of PWID are Medicaid beneficiaries.^7^ Disparities in HCV treatment may differ across states, at least in part because of differences in Medicaid policies. Since 2014, some Medicaid programs have imposed requirements for documentation of sobriety, severe liver disease, or consultation with a specialist as a condition of covering DAA treatment.^8^ Though many states have removed these restrictions, 16 states and Puerto Rico still have 1 or more such requirements.^8^ These policies, to the extent that they may reduce treatment uptake, could be detrimental to the federal goal of HCV elimination.

Recently, the release of national administrative Medicaid data has enabled large scale analysis of care for this population. We examined disparities in the 6-month initiation of HCV treatment among Medicaid beneficiaries newly diagnosed with HCV. We chose this population to reflect incident HCV diagnoses, thus reflecting current and future disparities patterns, and chose a short-term outcome because of the importance of rapid treatment as a goal to prevent HCV transmission.

## Methods

This study was reviewed by the Biomedical Research Alliance of New York institutional review board, and consent was waived because of deidentified patient data. This study followed the Strengthening the Reporting of Observational Studies in Epidemiology (STROBE) reporting guideline.

### Design and Data Source

We conducted a retrospective cohort study using data from the 2017 to 2019 Transformed Medicaid Statistical Information System Analytic File, which contains insurance claims and encounter data for all Medicaid beneficiaries in all states and territories, including those enrolled in both fee-for-service and managed care programs. We used data from 47 states, Washington DC, and Puerto Rico; data from Rhode Island, Tennessee, and Kansas were omitted because they were missing race and ethnicity data for more than 50% of sampled individuals.

### Study Population

We extracted health care claims of individuals with a new diagnosis of HCV infection. HCV is diagnosed through laboratory test results, which are not available in health insurance claims data. We adapted an algorithm validated by Isenhour et al,^9^ which requires a *Current Procedural Terminology* (*CPT*) code for an HCV RNA test followed by an *International Statistical Classification of Diseases and Related Health Problems, Tenth Revision *(*ICD-10*) code for HCV within 180 days. We required a 1 year look-back period prior to the *ICD-10* diagnosis, in which a RNA test and *ICD-10* combination was not observed. The index date was the date on which the *ICD-10* appeared. We included participants who were ages 18 to 64 years at the index month and were continuously enrolled in Medicaid (and not dually enrolled in Medicare) for 12 months prior to and 6 months after the index month.

### Study Outcome

The primary outcome was DAA treatment initiation, defined by a filled prescription for DAAs up to 180 days after the date of diagnosis. We chose a relatively short interval because (1) treatment is recommended for all individuals at the time of diagnosis; (2) the case definition, which requires an HCV RNA test followed by an *ICD* diagnostic code, already accounts for the lag time between testing, diagnosis, and awareness of the diagnosis by health care professionals; and (3) this mitigates the amount of sample attrition associated with Medicaid disenrollment.^10^ DAA prescription was identified based on National Drug Codes (NDCs) in pharmacy claims (eTable 1 in Supplement 1).

### Exposure Variables

We examined categorical age, sex, and race and ethnicity as reported in the data set and collected by each state’s programs from beneficiaries at enrollment without recategorization. If race or ethnicity was missing for any beneficiary in the year and state of their index diagnosis, we attempted to fill the missing value if the same beneficiary had a nonmissing value in other states or years. We interpreted race and ethnicity as a social and cultural construct and a proxy for structural racism, and thus considered it a key factor to include in this study because people of different racial and ethnic groups may have differential access to HCV treatment. We defined comorbidities using *ICD-10* codes in the 12-month lookback period, including pregnancy or postpartum status, IDU, alcohol use disorder, cirrhosis, mental health diagnoses, HIV, and hepatitis B (eTable 1 in Supplement 1). IDU was measured based on a previously validated algorithm for identifying past-year IDU that combines diagnosis codes for use, dependence, poisoning for injectable drugs, prescriptions for buprenorphine treatment, and codes for methadone treatment.^11^ Urbanicity was classified using the zip code–level 2010 Rural Urban Commuting Area Codes in 4 categories.^12^ We assigned each zip code to a decile of median family income, based on data from the American Community Survey.

### Sensitivity Analyses

We conducted 2 preplanned sensitivity analyses. Because temporary disenrollment is common in Medicaid, we relaxed the enrollment criteria by requiring that individuals were enrolled for more than 1 day in each month (ie, not necessarily the entire month).^13^ Also, recognizing that individuals who had an RNA test for HCV and a diagnostic code may have greater health care access than patients with only a diagnosis code, we conducted analysis using a more inclusive sample of all individuals receiving an HCV diagnosis code without a diagnosis code in the prior 12 months, regardless of whether they received an RNA test.

### Statistical Analysis

Statistical analyses were conducted using Stata version 17.0 (StataCorp). We conducted univariate analyses for all independent variables with the outcome of treatment initiation within 6 months, using χ^2^ test accounting for state-level clustering. We estimated multivariable logistic regression models for treatment initiation, adjusting for individual- and area-level variables conceptually associated with treatment initiation. We report odds ratios (OR) and 95% CIs, also accounting for state-level clustering using the clustered sandwich estimator. Statistical significance was set at *P* < .05. We also report adjusted differences in the treatment rate associated with each category of a covariate relative to the reference category, while holding other covariates at their observed values.

We examined possible between-state vs within-state differences in 2 ways. First, we added to our regressions dichotomous indicators of a given patient’s residential state (or other jurisdiction, in the case of Washington DC and Puerto Rico) at the time of the index month (ie, state fixed effects). This approach controls for time-invariant linear trends within each state. We examined how estimated disparities changed after this adjustment.

Because we expected that between-state differences may be driven by Medicaid prior authorization policies in this time period, we specifically controlled for these policies as a second approach. If disparities were largely driven by these policies, we would expect to see attenuated associations between the other covariates and treatment uptake. Otherwise, the results would indicate that other unmeasured factors were driving the between-state differences. We used results of cross-sectional policy surveys conducted in 2018 and 2019 by the National Viral Hepatitis Roundtable that cataloged fee-for-service Medicaid programs’ prior authorization requirements for DAA treatment.^8^ The 3 policy variables included need for documentation of liver fibrosis (fibrosis policy), need for documentation of either counseling about substance use or abstinence (sobriety policy), and restrictions on the specialty of the DAA prescriber (prescriber policy). Each policy was assigned as a time-varying variable, based on the policy in place in the patient’s state of residence in the year of their index diagnosis. Policies for fibrosis and prescribers were categorized as any restriction vs none. We expanded categorization of the sobriety policy to include required abstinence as a separate category from states requiring only counseling, as abstinence was a substantially more restrictive requirement.

## Results

Of 161 623 patients with an HCV diagnosis during the study period, 87 652 were included in the final analysis (eFigure in Supplement 1). Of the included patients, 43 078 (49%) were females, 12 355 (14%) were age 18 to 29 years, 35 181 (40%) were age 30 to 49 years, 51 282 (46%) were non-Hispanic White, and 48 840 (49%) had an IDU diagnosis. Additionally, 17 927 patients (20%) received HCV treatment within 6 months of their HCV diagnosis. Patients who initiated HCV treatment were more likely to be older, male, and less likely to have IDU (Table 1).

**Table 1.** Frequency of HCV DAA Treatment Uptake by 6 Months After Diagnosis

Characteristic	Patients, No. (%)		*P* value^a^
	Not treated (n = 69 725)	Treated (n = 17 927)	
Age category, y			
18-29	10 675 (15)	1680 (9)	<.001
30-49	28 447 (41)	6734 (38)	
50-64	30 603 (44)	9513 (53)	
Sex			
Male	34 237 (49)	10 337 (58)	<.001
Female	35 488 (51)	7590 (42)	
Race or ethnicity			
American Indian or Alaska Native	1331 (2)	248 (1)	.09
Asian	1170 (2)	236 (1)	
Hispanic	7650 (11)	2020 (11)	
Multiple	115 (<1)	24 (<1)	
Native Hawaiian or Other Pacific Islander	138 (<1)	28 (<1)	
Non-Hispanic White	40 912 (59)	10 370 (58)	
Non-Hispanic Black	10 509 (15)	2888 (16)	
Missing	7900 (11)	2113 (12)	
Injection drug use			
No	29 875 (43)	8937 (50)	.005
Yes	39 850 (57)	8990 (50)	
Cirrhosis			
No	64 753 (93)	16 403 (91)	.08
Yes	4972 (7)	1524 (9)	
Alcohol use disorder			
No	58 324 (84)	15 213 (85)	.13
Yes	11 341 (16)	2714 (15)	
Mental health diagnosis			
No	35 265 (51)	9775 (55)	.001
Yes	34 460 (49)	8152 (45)	
HIV			
No	66 250 (95)	17 215 (96)	.03
Yes	3475 (5)	712 (4)	
HBV			
No	68 842 (99)	17 755 (99)	.001
Yes	883 (1)	172 (1)	
Pregnancy or postpartum			
No	64 491 (92)	17 568 (98)	
Yes	5234 (8)	359 (2)	
Urbanicity (zip code–level RUCA2)^b^			
Urban	56 354 (81)	14 875 (83)	.25
Large rural	7221 (10)	1611 (9)	
Small rural	3531 (5)	808 (5)	
Isolated	2137 (3)	518 (3)	
Median family income, % of FPL^c^			
<200	20 902 (30)	4919 (27)	.004
200-300	30 643 (44)	7522 (42)	
300-400	10 906 (16)	3255 (18)	
>400	6141 (9)	1932 (11)	

Abbreviations: DAA, direct acting antiviral; FPL, Federal Poverty Level; HBV, hepatitis B virus; HCV, hepatitis C virus; RUCA2, Rural Urban Commuting Area version 2.

^a^ *P* values accounting for clustering of patients within states. Comorbidities based on *International Classification of Diseases, Ninth* and *Tenth Revisions*, Healthcare Common Procedure Coding System, and national drug codes.

^b^ Urbanicity from Rural-Urban Commuting Area codes.

^c^ Median family income from American Community Survey at zip code–level, categorized based on 2018 federal poverty level for a household of 4.

Table 2 shows the results of multivariable analyses. In model 1, we adjusted for demographics, comorbidities, and zip-code level income and rurality. We found significantly higher odds of DAA treatment initiation for males (OR, 1.24; 95% CI, 1.16-1.33; *P* < .001), and lower odds for younger age groups (OR, 0.65; 95% CI, 0.50-0.85; *P* = .001) and people with IDU diagnoses (OR, 0.84; 95% CI, 0.75-0.94; *P* = .002) as well as other comorbidities. We also found significantly lower odds of treatment initiation for Asian vs non-Hispanic White (OR, 0.62; 95% CI, 0.50-0.78; *P* < .001) and Native Hawaiian or Other Pacific Islander (OR, 0.66; 95% CI, 0.44-0.97; *P* = .04). Pregnancy or postpartum status was associated with lower treatment (OR, 0.34; 95% CI, 0.23-0.37; *P* < .001).

**Table 2.** Multivariable Regression of DAA Treatment Uptake Within 6 Months of New HCV Diagnosis

Characteristics	Model 1 (without state fixed effects)^a^			Model 2 (with state fixed effects)^a^		
	aOR (95% CI)	*P* value	Adjusted difference, %^b^	aOR (95% CI)	*P* value	Adjusted difference, %^b^
Age category, y						
18-29 y	0.65 (0.50-0.85)	.001	−6.5	0.66 (0.53-0.83)	<.001	−6.0
30-49 y	0.85 (0.74-0.98)	.03	−2.6	0.86 (0.75-0.98)	.03	−2.4
50-64	1 [Reference]	NA	Reference	1 [Reference]	NA	Reference
Sex						
Male	1.24 (1.16-1.33)	<.001	−3.5	1.24 (1.16-1.32)	<.001	−3.3
Female	1 [Reference]	NA	[Reference]	1 [Reference]	NA	[Reference]
Race or ethnicity						
American Indian or Alaska Native	0.77 (0.56-1.05)	.09	−4.0	0.68 (0.55-0.84)	<.001	−5.5
Asian	0.62 (0.50-0.78)	<.001	−6.7	0.50 (0.40-0.64)	<.001	−8.9
Hispanic	0.94 (0.70-1.26)	.69	−0.03	0.81 (0.71-0.93)	.003	−3.0
Multiple	0.88 (0.59-1.32)	.55	−1.9	0.79 (0.60-1.04)	.09	−3.4
Native Hawaiian or Pacific Islander	0.66 (0.44-0.97)	.04	−6.1	0.71 (0.50-1.01)	.06	−4.9
Non-Hispanic Black	0.94 (0.82-1.09)	.44	−0.9	1.03 (0.95-1.11)	.54	−0.6
Non-Hispanic White	1 [Reference]	NA	[Reference]	1 [Reference]	NA	[Reference]
Missing	0.95 (0.76-1.21)	.71	−0.7	0.99 (0.93-1.06)	.83	−0.1
Injection drug use						
Yes	0.84 (0.75-0.94)	.002	−2.8	0.81 (0.75-0.87)	<.001	−3.3
No	1 [Reference]	NA	[Reference]	1 [Reference]	NA	[Reference]
Cirrhosis						
Yes	1.08 (0.93-1.26)	.97	1.2	1.08 (0.93-1.27)	.32	1.2
No	1 [Reference]	NA	[Reference]	1 [Reference]	NA	[Reference]
Alcohol use disorder						
Yes	0.90 (0.81-1.00)	.04	−1.7	0.87 (0.80-0.94)	.001	−2.0
No	1 [Reference]	NA	[Reference]	1 [Reference]	NA	[Reference]
Mental health diagnosis						
Yes	0.93 (0.89-0.97)	.004	−1.1	0.94 (0.90-0.99)	.03	−0.9
No	1 [Reference]	NA	[Reference]	1 [Reference]	NA	[Reference]
HIV						
Yes	0.72 (0.59-0.89)	0.003	−4.7	0.70 (0.55-0.88)	.002	−5.0
No	1 [Reference]	NA	[Reference]	1 [Reference]	NA	[Reference]
HBV						
Yes	0.74 (0.64-0.86)	<.001	−4.3	0.77 (0.64-0.93)	.007	−3.7
No	1 [Reference]	NA	[Reference]	1 [Reference]	NA	[Reference]
Pregnancy or postpartum						
Yes	0.34 (0.23-0.37)	<.001	−13	0.34 (0.30-0.41)	<.001	−12
No	1 [Reference]	NA	[Reference]	1 [Reference]	NA	[Reference]
Urbanicity (zip code–level RUCA2)						
Urban	1 [Reference]	NA	[Reference]	1 [Reference]	NA	[Reference]
Large rural	0.92 (0.77-1.10)	.37	−1.3	0.97 (0.90-1.06)	.53	−0.4
Small rural	0.97 (0.82-1.15)	.75	−0.4	0.98 (0.89-1.08)	.70	−0.3
Isolated	1.0 (0.82-1.29)	.79	0.5	0.98 (0.87-1.09)	.67	−0.4
Median family income, % of FPL						
<200	1 [Reference]	NA	[Reference]	1 [Reference]	NA	[Reference]
200-300	1.08 (0.99-1.18)	.10	1.1	1.07 (1.02-1.13)	.004	1.1
300-400	1.31 (1.12-1.54)	.001	4.4	1.18 (1.09-1.28)	<.001	2.5
>400	1.37 (1.12-1.67)	.002	5.1	1.17 (1.04-1.33)	.009	2.5

Abbreviations: FPL, Federal Poverty Level; HBV, hepatitis B virus; NA, not applicable; RUCA2, Rural Urban Commuting Area version 2.

^a^ Both models use robust standard error for state-level clustering. Model 2 also adjusted for state identity as fixed-effect (not shown).

^b^ Adjusted difference in probability of treatment represents the difference in marginal estimated probability of treatment associated with the variable compared with the reference value.

In model 2, we additionally adjusted for state fixed effects. We found similar associations for sex, age, pregnancy or postpartum status, and comorbidities as in model 1. The crude treatment rates between Hispanic individuals and non-Hispanic White were not different (2020 of 9670 [20%] vs 10 370 of 51 282 [20%]). However, after adjusting for state fixed effects in model 2, we found a significantly lower odds of treatment initiation for Hispanic individuals (OR, 0.81; 95% CI, 0.71-0.93; *P* = .003) and American Indian or Alaska Native individuals (OR, 0.68; 95% CI, 0.55-0.84; *P* < .001). We found a larger magnitude of association for Asian individuals vs non-Hispanic White individuals when compared with the association seen in model 1 (OR, 0.50; 95% CI, 0.40-0.64; *P* < .001). In model 2, the association for Native Hawaiian or Other Pacific Islander individuals vs non-Hispanic White individuals was not significant.

In a separate regression analysis where we controlled for state-level policy characteristics instead of state fixed effects, the associations between each racial or ethnic group and treatment initiation were similar to those reported in model 2 (eTable 2 in Supplement 1). The results of sensitivity analyses were similar to the main analysis, except for changes in statistical significance of some estimates due to different population sizes (eTables 3 and 4 in Supplement 1).

## Discussion

In this national study of Medicaid enrollees, we found that the rate of HCV treatment initiation at 6 months was low overall, and significantly lower among individuals from some minoritized racial and ethnic groups, younger patients, females, and patients with IDU and other comorbidities. These findings suggest that substantial public health and policy efforts are needed to encourage HCV treatment and reduce disparities. Our data are among the first of which we are aware to comprehensively analyze HCV treatment in Medicaid, and reveal that even within this relatively disadvantaged population, inequities are present with significant public health consequences.

The overall rate of treatment initiation is low. Because HCV treatment supports HCV prevention by reducing risk of transmission, these findings may have dire consequences for HCV elimination planning, which relies on achieving high treatment rates. Two recent studies also found that few Medicaid enrollees with HCV infection are receiving timely treatment. Thompson et al^14^ found HCV treatment initiation among about 38 000 Medicaid beneficiaries with HCV-viremia was 23% within 1 year of diagnosis. Harris et al^15^ found an HCV treatment initiation rate of 30% among 27 000 Medicaid beneficiaries in selected states, despite following them up for a longer time. Both also found higher treatment among individuals who were commercially insured (68% in Harris et al^15^ and 35% in Thompson et al^14^).

Treatment initiation was significantly lower among younger individuals. Likewise, treatment was lower among people who had diagnoses or treatments suggestive of IDU. Young PWID are a priority population for elimination programming due to several barriers to treatment, including low perceived severity of HCV, its long asymptomatic period, decreased engagement with health care, stigma, socioeconomic instability, and policies that restrict treatment coverage to those with severe disease or documented sobriety. These findings point to the need to integrate HCV care into venues that offer services for young PWID, through colocated care or via telehealth.^16,17^ A recent study^18^ illustrated high patient satisfaction with telehealth encounters integrated into opioid treatment programs. The lower rate of treatment among females is also concerning, given the extent to which they comprise a large proportion of the youngest cohorts. This may be related to the absence of approved treatments for use in pregnancy. However, adjusting for pregnancy or postpartum status alone did not eliminate the gender disparity, suggesting that other systemic barriers to HCV treatment access may exist for females, such as increased stigma or decreased propensity for health care professionals to offer treatment.

The lower rate of treatment for individuals with cirrhosis and HIV was both surprising and concerning. For cirrhosis, the finding may be related to our definition of comorbid conditions as those present before the HCV diagnosis. In reality, many individuals would receive a workup and diagnosis for cirrhosis only after HCV is diagnosed. Individuals with preexisting cirrhosis diagnoses who subsequently are diagnosed with HCV may represent a unique clinical population, including individuals who are not eligible for or who have declined HCV treatment. Additional clinical data are needed to shed further light on this finding. Individuals with HIV coinfection also had lower likelihood of treatment. This may be due to many health care professionals’ discomfort with treating HIV and HCV coinfections, particularly with managing drug interactions, and barriers associated with referral to hepatology specialists.

Our findings suggest that while Medicaid prior authorization policies are an important factor, the elimination of these policies may not be enough to ensure equitable treatment uptake among individuals from minoritized racial and ethnic groups. Lower treatment initiation among Hispanic and American Indian or Alaska Native populations became apparent only after controlling for between-state differences and, specifically, for Medicaid policies. This suggests that there are within-state racial or ethnic disparities, which may be obscured in national data. For example, Hispanic individuals were concentrated in states that did not require sobriety for coverage of DAA, a policy that in turn was associated with increased treatment. Thus, significant disparities among Hispanic individuals were not observed at a national level but were present among individuals exposed to similar health coverage environments. Future studies that measure disparities among patients with HCV should be attentive to this possibility and attempt to control for differences in state-level factors. Additionally, states that have eliminated (or are planning to eliminate) these restrictions would still need to be attentive to racial or ethnic disparities in treatment initiation.

Our data showed lower treatment initiation among Asian individuals consistently across model specifications. The treatment access and experiences of Asian American individuals who are Medicaid beneficiaries are not well studied. Asian American individuals are less likely than other groups to receive treatment for substance use disorder, regardless of insurance.^19^ A particular challenge in understanding this disparity is the ethnic and socioeconomic heterogeneity of the Asian American population.^20^ For example, HCV in Asian American individuals may be particularly concentrated in people who emigrated from endemic countries, and who in turn may face a higher risk of decreased health care access due to limited English proficiency.^21,22^ Programs that are focused on hepatitis B virus prevention and testing in Asian American individuals may also be effective at closing the disparities observed with HCV. Detailed analysis with data sources that allow for disaggregation of factors that may influence health care access, including country of birth, language preference, and individual socioeconomic status, are needed to further characterize this disparity.

### Limitations

This study has limitations. HCV diagnosis relies on detection of HCV RNA, and the results of laboratory testing are not available in this data set. Testing that is performed without generation of a procedure code, for example through community-based programs or block grants, may not be reflected here. We can only report treatment disparities conditional on having a diagnosis in a health care setting. The true magnitude of treatment disparities may differ when accounting for differences in testing rates or health care access, given that many patients with HCV may be undiagnosed or are not linked to care.^23^ We also could not examine health care patterns of individuals with long-standing HCV, due to the short study period. *ICD-10* codes are used to define comorbidities, but these may be inaccurately or inconsistently reported. Codes related to substance use disorder do not differentiate between IDU and noninjection drug use; however, we attempted to mitigate this limitation by using a previously validated algorithm. Furthermore, there are significant missing race or ethnicity data, and we recognize that race and ethnicity as reported to Medicaid programs does not reflect the complexity of individuals’ identities. We attempted to minimize the impact of missing data on the results by accounting for nonmissing values across different states and years and by excluding states with high levels of missingness. Though the mechanism of missing race or ethnicity data is not known, there are no associations between missing race and treatment in any model specification. We reported disaggregated data to the extent we were able, but a consequence of doing so is that some groups with smaller sample sizes have low precision in regression analyses.

## Conclusions

In this cohort study, we found that the rate of HCV treatment among patients with a new diagnosis of HCV in Medicaid administrative data are low and that there are significant disparities among the highest priority groups for HCV elimination—young people, PWID, and females. The observed racial and ethnic disparities in treatment initiation suggest that continued tracking of these inequities, and understanding of their mechanisms, are needed. Further interventions to improve treatment uptake are also needed in the key populations identified here, in order to achieve the treatment rates needed to eliminate HCV.

## Notes

Supplement 1. **eTable 1.** National Drug Codes (NDCs), *International Classification of Disease, 10th Revision *(*ICD-10*) and Current Procedure Terminology (CPT) Codes Used **eFigure 1.** Diagram of Patients Included and Excluded From Analysis **eTable 2.** Race/Ethnicity Associated With Treatment After Adjustment for Policy Categories **eTable 3.** Sensitivity Analysis 1 **eTable 4.** Sensitivity Analysis 2

Supplement 2.
Data Sharing Statement
