# Supplementary material for: Hepatitis C Treatment Initiation Among US Medicaid Enrollees
Source: JAMA Netw Open. 2023 Aug 4;6(8):e2327326. doi: 10.1001/jamanetworkopen.2023.27326 (PMC10403776; doi:10.1001/jamanetworkopen.2023.27326)
Supplement: Supplement 1. — eTable 1. National Drug Codes (NDCs), International Classification of Disease, 10th Revision (ICD-10) and Current Procedure Terminology (CPT) Codes Used eFigure 1. Diagram of Patients Included and Excluded From Analysis eTable 2. Race/Ethnicity Associated With Treatment After Adjustment for Policy Categories eTable 3. Sensitivity Analysis 1 eTable 4. Sensitivity Analysis 2 [file jamanetwopen-e2327326-s001.pdf]

Supplemental Online Content

Kapadia SN, Zhang H, Gonzalez CJ, et al. Hepatitis C treatment initiation among US Medicaid enrollees. *JAMA Netw Open*. 2023;6(8):e2327326. doi:10.1001/jamanetworkopen.2023.27326

eTable 1. National Drug Codes (NDCs), *International Classification of Disease, 10th Revision* (*ICD-10*) and Current Procedure Terminology (CPT) Codes Used
eFigure 1. Diagram of Patients Included and Excluded From Analysis
eTable 2. Race/Ethnicity Associated With Treatment After Adjustment for Policy Categories
eTable 3. Sensitivity Analysis 1
eTable 4. Sensitivity Analysis 2

This supplemental material has been provided by the authors to give readers additional information about their work.

© 2023 Kapadia SN et al. *JAMA Network Open.*

eTable 1.

National Drug Codes (NDCs), International Classification of Disease, 10th Revision (ICD-10) and Current Procedure Terminology (CPT) codes used

Variable	Type	Code List
HCV RNA Test	CPT	87520, 87521, 87522
HCV Diagnosis	ICD-10	B17.10; B17.11; B18.2; Z22.52; B19.20; B19.21
HCV Direct Acting Antiviral Treatment	NDC	61958-2201-1; 61958-2203-1; 61958-1801-1; 61958-1803-1; 61958-1804-1; 61958-1805-1; 72626-2601-1; 72626-2701-1; 61958-1501-1; 61958-1503-1; 61958-1504-1; 61958-1505-1; 61958-2401-1; 0074-2625-01; 0074-2625-28; 0074-2625-56; 0006-3074-02; 0003-0011-01; 0003-0213-01; 0003-0215-01; 0074-0063-01; 0074-0063-28; 0074-3093-01; 0074-3093-28; 0074-3082-28; 59676-225-07; 59676-225-28;
Injection drug use-related diagnoses	ICD-10	F11.X; F13.X; F14.X; F15.X; F19.X; R78.1X; R78.2X; T38.7X; T40.0X; T40.1X; T40.2X; T40.3X; T40.4X; T40.5X; T40.6X; T40.8X; T40.9X; T41.2X; T42.X; T43.6X; T43.8X; T43.9X; T50.7X; Z50.3X; Z71.5X; 969.9X; 970.X; V65.42
Buprenorphine Prescription	NDC	54017613; 54017713; 54018813; 54018913; 93360021; 93360040; 93360121; 93360140; 93360221; 93360240; 93360321; 93360340; 93365621; 93365640; 93365721; 93365740; 93365821; 93365840; 93365921; 93365940; 93537856; 93537956; 93572056; 93572156; 228315303; 228315403; 228315473; 228315503; 228315567; 228315573; 228315603; 378092393; 378092493; 378876593; 378876693; 378876716; 378876793; 378876816; 378876893; 406192303; 406192309; 406192403; 406192409; 406800503; 406802003; 490005100; 490005130; 490005160; 490005190; 781721664; 781722764; 781723806; 781723864; 781724964; 12496010001; 12496010002; 12496010005; 12496030001; 12496030002; 12496030005; 12496120201; 12496120203; 12496120401; 12496120403; 12496120801; 12496120803; 12496121201; 12496121203; 12496127802; 12496128302; 12496130602; 12496131002; 16590066605; 16590066630; 16590066705; 16590066730; 16590066790; 23490927003; 23490927006; 23490927009; 35356000407; 35356000430; 35356055530; 35356055630; 42291017430; 42291017530; 42858035340; 42858049340; 42858050103; 42858050203; 42858058640;

© 2023 Kapadia SN et al. *JAMA Network Open.*

Variable	Type	Code List
		42858075040; 42858083940; 43063018407; 43063018430; 43063066706; 43063075306; 43598057901; 43598057930; 43598058001; 43598058030; 43598058101; 43598058130; 43598058201; 43598058230; 47781035503; 47781035603; 47781035703; 47781035803; 47781071203; 47781071211; 49999039507; 49999039515; 49999039530; 49999063830; 49999063930; 50090157100; 50090292400; 50268014411; 50268014415; 50268014511; 50268014515; 50383028793; 50383029493; 50383092493; 50383093093; 52427069203; 52427069211; 52427069403; 52427069411; 52427069803; 52427069811; 52440010014; 52959030430; 52959074930; 53217013830; 53217024630; 54123011430; 54123090730; 54123091430; 54123092930; 54123095730; 54123098630; 54569549600; 54569573900; 54569573901; 54569573902; 54569639900; 54569640800; 54569657800; 54868570700; 54868570701; 54868570702; 54868570703; 54868570704; 54868575000; 55045378403; 55700014730; 55700018430; 55700030230; 55700030330; 55700056804; 55700057904; 55887031204; 55887031215; 58284010014; 59385001201; 59385001230; 59385001401; 59385001430; 59385001601; 59385001630; 60429058630; 60429058633; 60429058730; 60429058733; 60687048111; 60687048121; 60687049211; 60687049221; 60846097003; 60846097103; 62175045232; 62175045832; 62756045964; 62756045983; 62756046064; 62756046083; 62756096964; 62756096983; 62756097064; 62756097083; 63629403401; 63629403402; 63629403403; 63629409201; 63629409202; 63629507401; 63629712501; 63629712502; 63629712503; 63629712504; 63629712505; 63629712506; 63629712507; 63629712601; 63629712602; 63629712603; 63629712604; 63629712605; 63629712606; 63629712607; 63629712608; 63629727001; 63629727002; 63874108403; 63874108503; 63874117303; 63874117403; 64725093003; 64725093004; 64725192403; 64725192404; 65162041503; 65162041509; 65162041603; 65162041609; 66336001530; 66336001630; 68071138003; 68071151003; 68258299103;

© 2023 Kapadia SN et al. *JAMA Network Open.*

Variable	Type	Code List
		68258299903; 68308020230; 68308020830; 69238120202; 69238120302; 69238120402; 69238120502; 69238150502; 70518155700; 71335035301; 71335035302; 71335035303; 71335035304; 71335035305; 71335035306; 71335035307; 71335115403; 76519117000; 76519117001; 76519117002; 76519117003; 76519117004; 76519117005;
Methadone maintenance treatment	CPT	H0020
Alcohol Use Disorder	ICD-10	F10.X; T51.X; K86.0; I42.6; K29.2X; G62.1
Cirrhosis	ICD-10	K70.3; K74.0; K74.6
HBV	ICD-10	B16.1; B16.2; B18.0; B18.1
HIV	ICD-10	B20.X; B21.X; B22.X; B23.X; B24.X; Z21
**Mental Health Diagnoses**		
Dementias		F03.90, F05, F01.50, F01.51
Transient mental disorders due to conditions classified elsewhere		F05, F06.2, F06.0, F06.30, F06.4, F06.1, F53, F06.8
Persistent mental disorders due to conditions classified elsewhere		F04, F02.80, F02.81, F03.90, F03.91, F06.0, F06.8
Schizophrenic disorders		F20.89, F20.1, F20.2, F20.0, F20.81, F20.5, F25.9, F20.9
Episodic mood disorders		F30.10, F30.11, F30.12, F30.13, F30.2, F30.3, F30.4, F32.9, F32.0, F32.1, F32.2, F32.3, F32.4, F32.5, F33.9, F33.0, F33.1, F33.2, F33.3, F33.41, F33.42, F31.10, F31.11, F31.12, F31.13, F31.2, F31.73, F31.74, F31.30, F31.31, F31.32, F31.4, F31.5, F31.75, F31.76, F31.60, F31.61, F31.62, F31.63, F31.64, F31.77, F31.78, F31.9, F30.8, F32.89, F31.81, F39, F34.81, F34.89
Delusional disorders		F22, F24, F23
Other nonorganic psychoses		F32.3, F33.3, F28, F44.89, F23, F29
Anxiety, dissociative and somatoform disorders		F41.9, F41.0, F41.1, F41.8, F44.9, F44.4, F44.6, F44.0, F44.1, F44.81, F44.89, F68.11, F68.8, F40.9, F40.01, F40.10, F40.218, F40.240, F40.241, F40.8, F42.2, F42.3, F42.8, F42.9, F34.1, F48.8, F48.1, F45.21, F45.22, F45.0, F45.1, F45.9, F45.8, F48.9, F99
Personality disorders		F60.0, F34.0, F60.89, F34.1, F60.1, F21, F60.3, F60.5, F60.4, F68.12, F60.7, F60.2, F60.81, F60.6, F60.9
Sexual and gender identity disorders		F66, F65.89, F65.4, F65.1, F65.2, F64.0, Z87.890, F64.0, F64.2, R37, F52.0, F52.21, F52.8, F52.31, F52.32, F52.4, F52.6, F52.1, F65.0, F65.3, F65.51, F65.52, F64.1, F65.81, F66, F65.9

© 2023 Kapadia SN et al. *JAMA Network Open.*

Variable	Type	Code List
Physiological malfunction arising from mental factors		F45.8, F42.4, F52.5, F59, F45.9
Special symptoms or syndromes not elsewhere classified		F98.5, F50.00, F95.9, F95.0, F95.1, F95.2, F98.4, F51.9, F51.02, F51.09, F51.01, F51.03, F51.19, F51.11, F51.12, F51.8, F51.3, F50.9, F50.2, F98.3, F98.21, F50.89, F50.81, F50.82, F50.89, F98.29, F98.0, F98.1, F45.41, G44.209, F45.42, F63.3, R45.1
Acute reaction to stress		F43.0, R45.7
Adjustment reaction		F43.21, F93.0, F94.8, F43.22, F43.23, F43.29, F43.24, F43.25, F43.10, F43.12, F43.8, F43.20
Depressive disorder, not elsewhere classified		F32.9
Disturbance of conduct not elsewhere classified		F91.1, F91.8, F91.2, F63.9, F63.0, F63.2, F63.1, F63.81, F63.3, F63.89, F91.9
Disturbance of emotions specific to childhood and adolescence		F93.8, F94.0, F91.3, F94.1, F98.8, F93.9, F94.8, F98.9
Hyperkinetic syndrome of childhood		F90.0, F90.9, F90.1, F90.2, F90.8
Psychic factors associated with diseases classified elsewhere		F54

© 2023 Kapadia SN et al. *JAMA Network Open.*

eFigure 1: Diagram of patients included and excluded from analysis

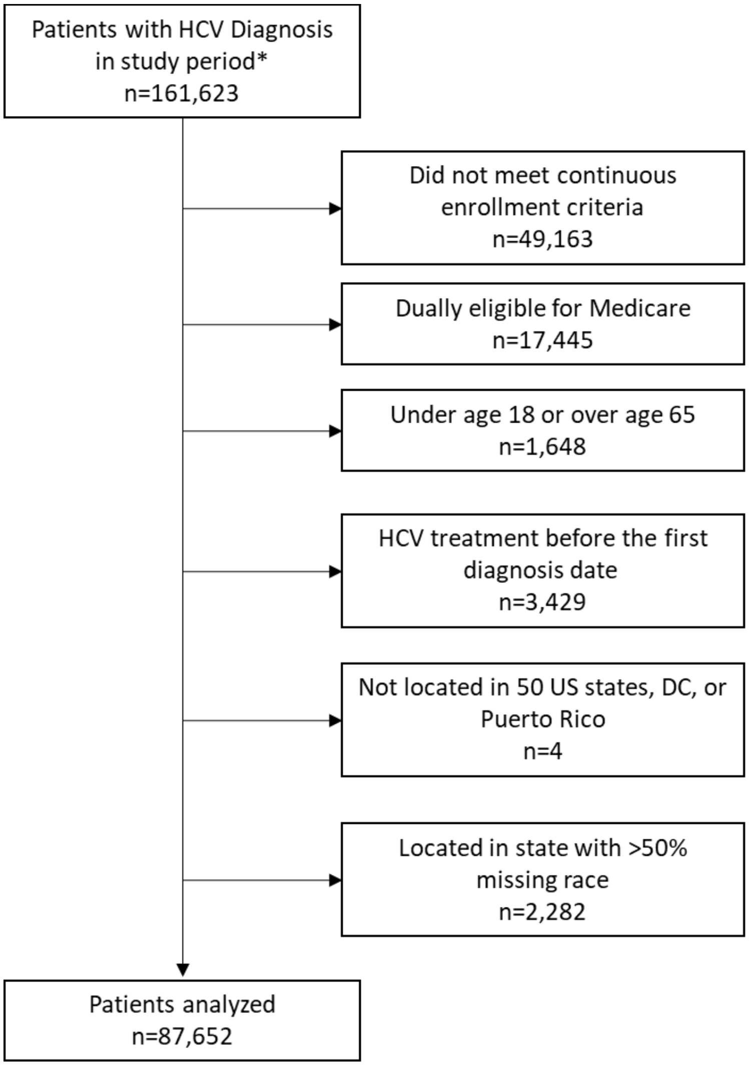

***HCV diagnosed defined as a CPT code for HCV RNA Testing followed by an ICD-10 code for HCV within 180 days.

© 2023 Kapadia SN et al. *JAMA Network Open.*

eTable 2: Race-ethnicity association with HCV treatment, with adjustment for state Medicaid policies and clinical + demographic factors

	aOR (95% CI)	p-val	Marginal effect
**Race/Ethnicity (ref=non-Hispanic White)**			
American Indian/Alaska Native	0.73 (0.55-0.97)	0.03	-4.6%
Asian	0.52 (0.43-0.62)	<0.001	-8.8%
Hispanic	0.83 (0.67-1.03)	0.10	-2.7%
Multiple	0.67 (0.50-0.90)	0.007	-5.7%
Native Hawaiian / Pacific Islander	0.71 (0.48-1.05)	0.09	-4.9%
Non-Hispanic Black	0.92 (0.84-1.01)	0.10	-1.3%
Missing	0.95 (0.83-1.08)	0.43	-0.9%
**Fibrosis Restrictions (ref=No restriction)**			
Any restrictions	0.78 (0.60-1.03)	0.08	-4%
**Sobriety Restrictions (Ref = No restriction)**			
Screening and counseling	1.00 (0.74-1.36)	0.99	+0.01%
Documented Abstinence	0.54 (0.32-0.92)	0.02	-9.3%
**Prescriber Restrictions (Ref = No restriction)**			
Any restriction	0.88 (0.65-1.19)	0.40	-2.0%

Caption: Standard errors are clustered by state. Model Also adjusted for age category, sex, injection drug use, cirrhosis, mental health, alcohol use disorder, HIV, HBV, pregnancy/postpartum, income range, rurality, and Medicaid Expansion status. Marginal effect represents the difference in marginal predicted probability of treatment associated with the variable compared to the reference value.

© 2023 Kapadia SN et al. *JAMA Network Open.*

eTable 3:

Sensitivity Analysis 1 : Multivariable Logistic Regression on outcome of DAA treatment initiation at 6-months among Medicaid enrollees with newly diagnosed HCV who were enrolled at least one day in each of the 12 months prior to and 6 months after their index diagnosis (n=89,734)

	**aOR (95% CI)**	**p-value**
**Age Category (ref=50-64 years)**		
18-29 years	0.67 (0.53-0.84)	<0.001
30-49 years	0.86 (0.75-0.98)	0.03
**Sex (ref=Female)**		
Male	1.24 (1.16-1.32)	<0.001
**Race/Ethnicity (ref=non-Hispanic White)**		
American Indian/Alaska Native	0.67 (0.54-0.83)	<0.001
Asian	0.50 (0.40-0.64)	<0.001
Hispanic	0.81 (0.71-0.93)	0.002
Multiple	0.83 (0.60-1.14)	0.25
Native Hawaiian / Pacific Islander	0.69 (0.49-0.97)	0.04
Non-Hispanic Black	1.03 (0.95-1.12)	0.64
Missing	0.99 (0.93-1.06)	0.85
**Injection Drug Use (ref=No)**		
Yes	0.81 (0.75-0.88)	<0.0001
**Cirrhosis (ref=No)**		
Yes	1.07 (0.92-1.25)	0.39
**Alcohol Use Disorder (ref=No)**		
Yes	0.87 (0.80-0.94)	0.001
**Mental Health Diagnosis (ref=No)**		
Yes	0.94 (0.89-0.99)	0.02
**HIV (ref=No)**		
Yes	0.69 (0.55-0.88)	0.002
**HBV (ref=No)**		
Yes	0.76 (0.63-0.93)	0.007
**Pregnancy/Postpartum (ref=No)**		
Yes	0.35 (0.30-0.41)	<0.001
**Urbanicity (ZIP-code level RUCA2)[b] (ref = Urban)**		
Large Rural	0.97 (0.89-1.05)	0.42
Small Rural	0.98 (0.88-1.08)	0.62
Isolated	0.98 (0.87-1.09)	0.67
**Median Family Income (ref = <200% of FPL)**		
200-300% of FPL	1.08 (1.03-1.05)	0.002
300-400% of FPL	1.19 (1.10-1.29)	<0.001
>400% of FPL	1.17 (1.03-1.33)	0.014

Caption: Also adjusted for state identity as fixed-effect (not shown). Abbreviations: HIV = human immunodeficiency virus, HBV = hepatitis B virus, RUCA2 = Rural Urban Commuting Area version 2. FPL = Federal Poverty Level in 2018 for family of 4.

© 2023 Kapadia SN et al. *JAMA Network Open.*

eTable 4:

Sensitivity Analysis 2 : Multivariate Logistic Regression on outcome of DAA treatment initiation at 6-months among Medicaid enrollees with an ICD-code diagnosis of HCV, regardless of whether they had a preceding RNA test. (n=184,085)

	**aOR (95% CI)**	**p-value**
**Age Category (ref=50-64 years)**		
18-29 years	0.79 (0.65-0.95)	0.02
30-49 years	0.92 (0.82-1.04)	0.20
**Sex (ref=Female)**		
Male	1.28 (1.21-1.36)	<0.001
**Race/Ethnicity (ref=non-Hispanic White)**		
American Indian/Alaska Native	0.82 (0.71-0.95)	0.007
Asian	0.42 (0.34-0.52)	<0.001
Hispanic	0.80 (0.71-0.90)	<0.001
Multiple	0.73 (0.62-0.88)	<0.001
Native Hawaiian / Pacific Islander	0.77 (0.51-1.16)	0.21
Non-Hispanic Black	0.96 (0.89-1.04)	0.34
Missing	0.89 (0.82-0.98)	0.02
**Injection Drug Use (ref=No)**		
Yes	0.70 (0.62-0.80)	<0.001
**Cirrhosis (ref=No)**		
Yes	0.75 (0.67-0.84)	<0.001
**Alcohol Use Disorder (ref=No)**		
Yes	0.69 (0.63-0.76)	<0.001
**Mental Health Diagnosis (ref=No)**		
Yes	0.85 (0.79-0.90)	<0.001
**HIV (ref=No)**		
Yes	0.61 (0.49-0.76)	<0.001
**HBV (ref=No)**		
Yes	0.19 (0.10-0.38)	<0.001
**Pregnancy/Postpartum (ref=No)**		
Yes	0.32 (0.27-0.37)	<0.001
**Urbanicity (ZIP-code level RUCA2)[b] (ref = Urban)**		
Large Rural	1.06 (0.95-1.18)	0.31
Small Rural	1.05 (0.97-1.15)	0.21
Isolated	1.09 (0.95-1.25)	0.23
**Median Family Income (ref = <200% of FPL)**		
200-300% of FPL	1.10 (1.04-1.15)	<0.001
300-400% of FPL	1.21 (1.11-1.32)	<0.001
>400% of FPL	1.24 (1.10-1.38)	<0.001

Caption: Also adjusted for state identity as fixed-effect (not shown). Abbreviations: HIV = human immunodeficiency virus, HBV = hepatitis B virus, RUCA2 = Rural Urban Commuting Area version 2. FPL = Federal Poverty Level in 2018 for family of 4.

© 2023 Kapadia SN et al. *JAMA Network Open.*
