# Supplementary material for: Hepatitis C Treatment Initiation Among US Medicaid Enrollees
Source: JAMA Netw Open. 2023 Aug 4;6(8):e2327326. doi: 10.1001/jamanetworkopen.2023.27326 (PMC10403776; doi:10.1001/jamanetworkopen.2023.27326)
Supplement: Supplement 2. — Data Sharing Statement [file jamanetwopen-e2327326-s002.pdf]

Data Sharing Statement

Kapadia. Hepatitis C Treatment Initiation Among US Medicaid Enrollees. *JAMA Netw Open*. Published August 04, 2023. doi:10.1001/jamanetworkopen.2023.27326

Data
Data available: No

Additional Information
Explanation for why data not available: The data is governed by a DUA with Centers for Medicare and Medicaid Services prohibiting us from sharing individual-level data. However, the dataset used for this study is available for purchase from Centers for Medicare and Medicaid Services
